# Contribution of the Hair Follicular Pathway to Total Skin Permeation of Topically Applied and Exposed Chemicals

**DOI:** 10.3390/pharmaceutics8040032

**Published:** 2016-11-15

**Authors:** Fadli Mohd, Hiroaki Todo, Masato Yoshimoto, Eddy Yusuf, Kenji Sugibayashi

**Affiliations:** 1 School of Pharmacy, Management & Science University, University Drive, Shah Alam 40100, Malaysia; m_fadli@msu.edu.my (F.M.); eddy@msu.edu.my (E.Y.); 2 Faculty of Pharmaceutical Sciences, Josai University, Saitama 350-0295, Japan; ht-todo@josai.ac.jp (H.T.); dotai@josai.ac.jp (M.Y.)

**Keywords:** hair follicle contribution, skin permeation, hydrophilic drugs, hair follicle plugging, transdermal delivery

## Abstract

Generally, the blood and skin concentration profiles and steady-state skin concentration of topically applied or exposed chemicals can be calculated from the in vitro skin permeation profile. However, these calculation methods are particularly applicable to chemicals for which the main pathway is via the stratum corneum. If the contribution of hair follicles to the total skin permeation of chemicals can be obtained in detail, their blood and skin concentrations can be more precisely predicted. In the present study, the contribution of the hair follicle pathway to the skin permeation of topically applied or exposed chemicals was calculated from the difference between their permeability coefficients through skin with and without hair follicle plugging, using an in vitro skin permeation experiment. The obtained results reveal that the contribution of the hair follicle pathway can be predicted by using the chemicals’ lipophilicity. For hydrophilic chemicals (logarithm of *n*-octanol/water partition coefficient (log *K*_o/w_) < 0), a greater reduction of permeation due to hair follicle plugging was observed than for lipophilic chemicals (log *K*_o/w_ ≥ 0). In addition, the ratio of this reduction was decreased with an increase in log *K*_o/w_. This consideration of the hair follicle pathway would be helpful to investigate the efficacy and safety of chemicals after topical application or exposure to them because skin permeation and disposition should vary among skins in different body sites due to differences in the density of hair follicles.

## 1. Introduction

The body is exposed to many chemical compounds in daily life. These chemicals are mainly absorbed via oral, pulmonary and dermal routes, as well as through other mucosa. Among these, the dermal pathway is more easily accessed by chemicals than the other pathways because skin is the outermost tissue covering the whole body and has a large surface area [1]. The skin is also focused on as the site of application of drugs and cosmetics. The pathway for the permeation of therapeutic and cosmeceutical chemicals through the skin is thus very important to evaluate their effects. In the case of either skin application or skin exposure, skin permeation and the concentration of chemicals in skin should be investigated to evaluate their effects and/or toxicities.

Skin can be histologically divided into three different layers from the surface to deeper tissues: stratum corneum, viable epidermis, and dermis. The superficial layer, the stratum corneum, is composed of dead corneocytes embedded in intercellular lipid matrices consisting of ceramides, free fatty acids, cholesterol and cholesteryl esters [2]. These lipids are organized into lamellar structures in the intracellular region of the stratum corneum and form the primary barrier against the elimination of endogenous compounds and the penetration of exogenous chemicals through the skin [3]. The permeation profile of chemicals through the skin is theoretically expressed by Fick’s second law of diffusion, which expresses the behavior of chemicals passing through the stratum corneum [4,5]. On the other hand, many reports [6,7,8] have been published describing how skin appendages such as hair follicles and sweat glands are an important permeation/penetration pathway, especially for hydrophilic compounds and macromolecules. Many researchers have already investigated the transfollicular permeation of topically applied chemicals. Feldman et al. [9] and Maibach et al. [10] have reported that regional variation in percutaneous absorption occurred due to a difference in hair density. Hueber et al. [11] investigated the role of hair follicles as a skin permeation route with burn scar tissue. Grice et al. [12] studied the effect of drug uptake into hair follicles on skin permeation by a cyanoacrylate casting method. Silvia et al. [13] showed a differential stripping technique consisting of a tape-stripping phase followed by a cyanoacrylate biopsy for quantitative drug analysis from hair follicles. Hairy and non-hairy guinea pig skins were used for in vivo and in vitro studies to check the transfollicular absorption. Furthermore, pharmacokinetic modeling was also applied to define the relative contribution of the hair follicle route. In addition to these skin permeation studies, the diffusion pathway and the distribution of topically applied or exposed chemicals through and in skin were identified by imaging analysis using a confocal microscope [14,15,16]. However, none of these studies involved quantitative analyses, and few studies evaluated the contribution of the hair follicle pathway for topically applied or exposed chemicals by in vivo or in vitro skin permeation experiments [13,17,18,19,20]. We have already established a method for hair follicle plugging using cyanoacrylate-grease mixture. We then reported [21] that the permeation of hydrophilic chemicals through hair follicle-plugged skin was dramatically decreased, whereas lipophilic chemical permeation through hair follicle-plugged skin was seldom changed compared with that through non-hair follicle-plugged skin. Otberg et al. [22] applied caffeine in a mixed solution of ethanol:polylene glycol (30:70 *v*/*v*) to volunteers before and after blocking all hair follicles with a varnish solution at the site of its application. In terms of the results, caffeine was observed in blood 20 min after application on the hair follicle-blocked skin, but 5 min after topical application to normal skin. A possible reason for the more rapid appearance of caffeine in blood is the rapid absorption of the substance penetrating through hair follicles to blood capillaries. In addition, Trauer et al. [23] reported that the in vivo absorption of caffeine from whole skin was much more rapid and substantial than the in vitro absorption. Furthermore, a sandwich technique has been used to clarify the contribution of the hair follicle pathway to the whole absorption of topically applied chemicals [19]. The sandwich technique revealed that the hair follicle pathway could contribute 34% to 60% of the whole skin permeation of chemicals with different lipophilicities (log *K*_o/w_ range: −1.05 to 2.29) and molecular weights (*M*_W_ range: 251 to 362). However, no clear relationship has been reported between physicochemical properties and the contribution of the hair follicle pathway to whole skin permeation.

Blood and skin concentrations of topically applied or exposed chemicals can be calculated from the in vitro skin permeation profile by taking into consideration skin thickness [24] and the applied chemical concentrations. This is very important for evaluating the usefulness and safety of topically applied or exposed chemicals because pharmacodynamics and toxicodynamics can be expressed as functions of these concentrations. Thus, the permeation pathway of hydrophilic chemicals, namely, the stratum corneum and hair follicles, should be discussed in detail for each chemical.

The hair follicular openings occupy only about 0.1% of the whole skin area, but this can be as high as about 10% for the face around the mouth and scalp [7]. Thus, we have to take this into account in terms of the chemicals applied and the sites of exposure or application to skin in order to evaluate the usefulness and safety of chemicals. In the present study, the contribution of the hair follicle pathway to the permeation of topically applied or exposed chemicals was determined from the difference between the permeation coefficients of chemicals through skin with and without hair follicle plugging.

## 2. Methods

### 2.1. Materials

Lidocaine hydrochloride (LC), fluorescein isothiocyanate-dextran 4 kDa (FD-4), calcein sodium salt (Ca-Na) and ibuprofen (IP) were obtained from Sigma-Aldrich Co., Ltd. (St. Louis, MO, USA). Isosorbide dinitrate (ISDN) was kindly donated by Toko Pharmaceutical Industrial Co., Ltd. (Tokyo, Japan). Butyl paraben (BP) and isosorbide mononitrate (ISMN) were obtained from Tokyo Kasei Kogyo Co., Ltd. (Tokyo, Japan). Nile red was obtained from Kanto Chemical Co., Inc. (Tokyo, Japan). Fluorescein sodium salt (FL-Na), aminopyrine (AMP) and diisopropyl fluorophosphate (DFP) were obtained from Wako Pure Chemical Ind., Ltd. (Osaka, Japan). All other reagents and solvents were of reagent grade or HPLC grade, and were used without further purification. Table 1 shows the physicochemical properties of the model chemicals used in the present skin permeation experiment.

### 2.2. Determination of n-Octanol/Buffer Coefficient

*n*-Octanol was saturated with pH 3.0 citrate buffer, pH 5.0 citrate buffer, pH 7.4 phosphate buffer or pH 10.0 carbonate buffer for at least 24 h before the experiment at 37 °C. Drug was dissolved in *n*-octanol-saturated buffered solution. The obtained solution was mixed with an equal volume of buffer solution-saturated octanol at 37 °C for 24 h. Drug concentration in the aqueous phase was then analyzed by HPLC. Apparent *n*-octanol/buffer solution coefficients (*K*_o/w_) were determined.

### 2.3. Animals

Frozen pig ear skins were purchased from the National Federation of Agricultural Cooperative Associations (Tokyo, Japan). These skins were stored at −80 °C until the skin permeation experiments. All animal studies were carried out in line with the recommendations of the Institutional Board for Animal Studies, Josai University (Sakado, Saitama, Japan).

### 2.4. Preparation of Skin Membrane

Purchased skin was maintained frozen at −80 °C prior to use. The skin was thawed at room temperature and excised from the outer surface of pig ear after being cleaned with pH 7.4 phosphate-buffered saline (PBS). Excess fat was carefully removed and no cannel-like diffusion pathway by the removal process was visibly confirmed. The excised skin was mounted on a Franz-type diffusion cell to conduct the skin permeation study. In the case of skin permeation study with hair follicle-plugged skin, the plugging procedure was carried out after skin excision. The integrity of the excised skin was measured using a skin impedance meter 1 h after hydration with 1 mL of PBS and all of the skin samples used in the present study showed resistance of above 20 Kohms/cm^2^.

### 2.5. Hair Follicle-Plugging Process

**Figure 1:**
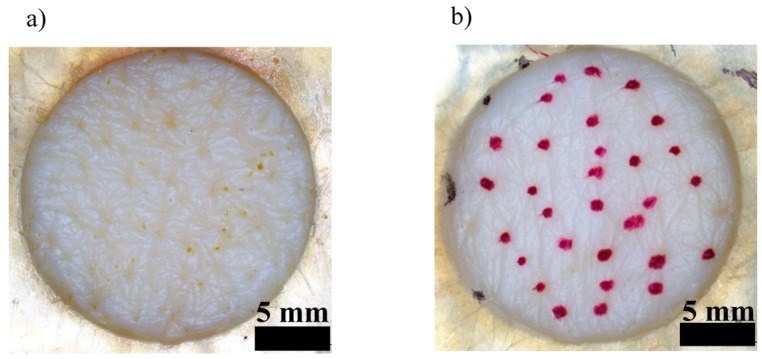
Pictures of the skin surface with (**a**) or without (**b**) hair follicle plugging.

The hair follicle-plugging procedure was as previously reported in detail [21]. This procedure can be briefly summarized as follows. Hair follicles in the designated area (effective skin permeation area: 1.77 cm^2^) were plugged with silicone grease-cyanoacrylate adhesive mixture paste to block chemical penetration through the hair follicles. The mixture paste consisted of equal parts of silicone grease (Super Lube^®^ Silicone Dielectric Grease; Synco Chemical Corp., Bohemia, NY, USA) and α-cyanoacrylate adhesives (Aron Alpha Jelly; Konishi Co., Ltd., Osaka, Japan) with small amounts of Nile red. Nile red was used to visualize the area to which the mixture paste had been applied. Thus, the hair follicles were plugged with the mixture paste to prevent chemical penetration through the follicular pathway. A mean of 56.1 ± 2.5 hair follicles (*n* = 15) were found in the effective skin permeation area (1.77 cm^2^) in the preliminary experiment, and half of them were plugged with the mixture paste in the present experiment. In our previous experiment, an almost linear decrease in the skin permeation ratio was observed with an increase in the number of hair follicles plugged with the mixture paste 17). The mixture paste-treated area was measured using imaging software (cellSens, Olympus Corp., Tokyo, Japan), equipped with a stereoscopic microscope (SZ61, Olympus Corp., Tokyo, Japan). The effective skin permeation area was about 1.62 cm^2^ after treatment with the mixture paste. Figure 1 shows the skin surface with or without the hair follicle plugging.

### 2.6. Preparation of Applied Solution

First, 5.0 mM FD-4, 1.0 mM FL-Na, 5.0 mM ISDN, 10 mM non-ionized AMP, 5.0 mM ionized IP, 0.6 mM BP and 500 mM ISMN were prepared with 1/30 mM phosphate-buffered saline (PBS, pH 7.4). Then, 1.0 mM CA-Na was prepared with pH 7.4 PBS containing 1.0 mM EDTA-2Na. In addition, 10 mM non-ionized LC was prepared with 100 mM carbonate-bicarbonate buffer solution (CaB, pH 10.0) and 100 mM ionized LC was prepared with 100 mM citrate buffer solution (CB, pH 5.0). Furthermore, 100 mM ionized AMP and 0.5 mM non-ionized IP were prepared with pH 3.0 CB. The pH levels for the fluorescent compounds such as FD-4, CA-Na and FL-Na and weak electrolytes except for BP were adjusted to ensure that about 99% were in non-ionized or ionized form.

### 2.7. In Vitro Skin Permeation Experiments

Excised pig ear skin membrane was mounted on vertical-type diffusion cells (effective diffusion area: 1.77 cm^2^). The stratum corneum was hydrated for 1 h with pH-adjusted solution or PBS containing 2.7 μmol/mL DFP. The latter was used to prevent the metabolism of ester compounds during the skin permeation experiment. It has already been confirmed that the hydration procedures with DFP did not affect the skin permeation of the esters and their metabolites [26,33,34].

After the pre-hydration process, solution applied to the skin was completely removed from the diffusion cell and 1.0 mL of test chemical solution and 6 mL of pH-adjusted solution were applied to the donor and receiver cells, respectively. A total of 0.54 μmol/mL DFP was added on the dermis side when ester compounds were applied on the stratum corneum. The permeation experiments were performed at 32 °C, while the receiver solution was continuously stirred with a star-head-type magnetic stirrer. At predetermined times, an aliquot (0.5 mL) was withdrawn from the receiver solution and an identical volume of fresh solution was added to keep the volume constant. Each experiment was performed in three to four replicates.

### 2.8. Determination of FD-4 and FL

The concentrations of FL, CA and FD-4 in the samples were analyzed using a spectrofluorophotometer (RF 5300PC; Shimadzu, Kyoto, Japan) at excitation wavelengths of 480, 488 and 490 nm, and at fluorescent emission wavelengths of 535, 515 and 520 nm, respectively.

### 2.9. Determination of Drugs

Concentrations of drugs (LC, ISDN, AMP, IP, BP and ISMN) in the samples were determined using an HPLC system (Prominence; Shimadzu, Kyoto, Japan) equipped with a UV detector (SPD-M20A; Shimadzu, Kyoto, Japan). The drug samples (0.2 mL) were added to the same volume of acetonitrile for ISMN or acetonitrile containing internal standard (methylparaben for LC, butylparaben for ISDN, AMP and IP, and propylparaben for BP), and mixed with a vortex mixer. After centrifugation at 21,500× *g* and 4 °C for 5 min, 20 μL of the supernatant was injected into the HPLC system. Chromatographic separation was performed using an Inertsil-ODS-3 (5 μm, 150 × 4.6 mm^2^ i.d.; GL Science, Kyoto, Japan) at 40 °C. The mobile phase was 0.1% phosphoric acid containing 5 mM sodium 1-heptanesulfate/acetonitrile (70/30, *v*/*v*) for LC, water/acetonitrile (55/45, *v*/*v*) for ISDN, 0.1% phosphoric acid containing 5 mM sodium dodecyl sulfate/acetonitrile (30/70, *v*/*v*) for AMP, 0.1% phosphoric acid/acetonitrile (55/45, *v*/*v*) for ISMN and water/acetonitrile (90/10, *v*/*v*) for ISMN. The flow rate was adjusted to 1.0 mL/min and detection was performed at UV 220 nm (ISMN and ISDN), 230 nm (LC), 245 nm (AMP), 260 nm (BP) or 263 nm (IP).

### 2.10. Analysis of Permeation Parameters

Skin permeation parameters were calculated using time courses of the cumulative amounts of chemicals that permeated through a unit area of skin with or without hair follicle plugging. The steady-state flux (*J*, a steady state that was reached 6–10 h after starting the experiment) was estimated from the slope of the linear portion of the profile of the cumulative amount of chemical that permeated through a unit area of skin versus time, and the lag time (*t*_lag_) was calculated from the intercept on the time axis by extrapolation from the steady-state skin permeation profile. From *J* and the donor concentration (*C*_v_), the permeability coefficient (*P*) was calculated using Equation (1). The partition parameter (*KL*) and the diffusion parameter (*DL*^−2^) were then obtained from Equations (2) and (3) [35]:

(1) $$P=\frac{J}{C_{v}}$$

(2) $$t_{\mathrm{lag}}=\frac{L^{2}}{6D}$$

(3) $$P=KL\cdot DL^{-2}$$

where *L*, *D* and *K* are the thickness of the barrier membrane, the diffusion coefficient of chemicals in the membrane and the partition coefficient of chemicals into the membrane, respectively. *DL*^−2^ was obtained from Equation (2) and *KL* was calculated from *P* and *DL*^−2^ values according to Equation (2).

In addition, the ratio of reduction of *P* of topically applied chemicals due to hair follicle plugging was calculated using Equation (4).

(4) $$\mathrm{Reduction}\;\mathrm{ratio}=\left(\frac{P\;\mathrm{through}\;\mathrm{nonplugged}\;\mathrm{skin}-P\;\mathrm{through}\;\mathrm{plugged}\;\mathrm{skin}}{P\;\mathrm{through}\;\mathrm{nonplugged}\;\mathrm{skin}}\right)\times 100$$

Then, the contribution of the hair follicle pathway to the whole skin permeation of chemicals was obtained by doubling the reduction ratio shown in the figure, since plugging of 30 of the total of 60 hair follicles in the effective skin area had been carried out.

### 2.11. Statistical Analysis

Statistical analysis was performed using unpaired Student’s *t*-test and ANOVA. A *p* value of less than 0.05 was considered significant.

## 3. Results

**Figure 2:**
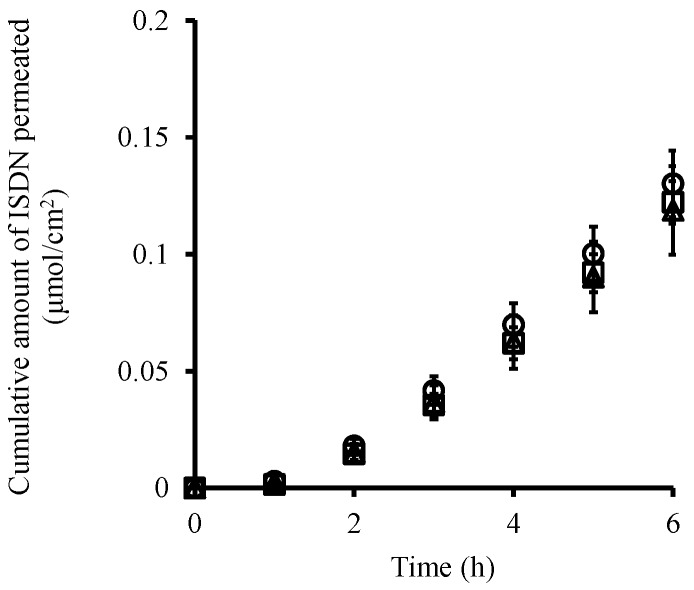
Time course of the cumulative amount of ISDN that permeated through pig ear skin from its solution. pH 3.0 (○), pH 7.4 (△) and pH 10.0 (□). Each point represents the mean ± S.E. (*n* = 3–4).

Figure 2 shows the time course of the cumulative amount of ISDN that permeated through pig ear skin from its solution with different pH levels (pH 3.0, pH 7.4 and pH 10.0) in order to investigate the effect of pH on the skin permeation of the neutral drug. As expected, almost the same ISDN permeation profiles were obtained independently of the pH under these experimental conditions. In addition, skin permeation parameters of *t*_lag_, *DL*^−2^ and *KL* obtained from the skin permeation profiles of ISDN for pH 3.0 and pH 10 solutions were very similar to those for the pH 7.4 solution (Table 2).

**Figure 3:**
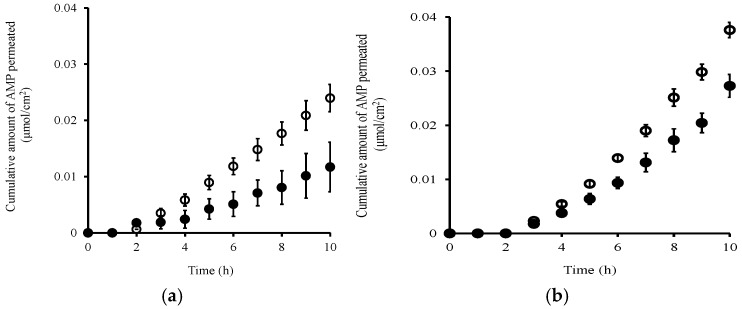
Time course of the cumulative amount of AMP that permeated through pig ear skin at pH 3.0 (**a**) and pH 7.4 (**b**). Non-hair-follicle-plugged skin (○), hair-follicle-plugged skin (●). Each point represents the mean ± S.E. (*n* = 4).

Figure 3a,b show the effect of hair follicle plugging on the skin permeation of ionized and non-ionized AMP, respectively. The cumulative amount of ionized AMP (at pH 3.0) that permeated through the plugged skin was about half of that through the non-plugged skin (Figure 3a). On the other hand, the cumulative amount of non-ionized AMP (at pH 7.4) that permeated through the plugged skin was about 0.7-fold that through the non-plugged skin (Figure 3b). The sizes of these decreases in the cumulative amount of AMP that permeated were greatly affected by the ratio of ionized to non-ionized forms. Table 3 shows permeation parameters calculated from the skin permeation profiles shown in Figure 3. These parameters can be utilized to evaluate the changes in distribution and/or diffusion of topically applied or exposed chemicals to and across the skin [8,36]. The *KL* values obtained through the hair follicle plugging were decreased at both pH 3.0 and pH 7.4 compared with those of non-plugged skin, although the *DL^−2^* values were almost the same. These decreased *KL* values at both pH 3.0 and pH 7.4 corresponded to the sizes of the decreases in the cumulative amount of AMP that permeated through the plugged skin compared with the non-plugged skin.

Next, a skin permeation experiment for several chemicals was carried out with or without hair follicle plugging to determine their *p* values as listed in Table 4. This table also shows the reduction ratio of *p* values calculated using Equation (2). Reductions of the *p* value of around 50% were observed for FD-4, Ca-Na, FL-Na, ionized LC, ionized AMP and ISMN, whereas no or only slight reductions (less than 20%) in *p* value were observed for non-ionized BP, non-ionized IP and non-ionized LC. Furthermore, reduction ratios of *p* values of 20% to 40% were shown for non-ionized AMP, ionized IP and ISDN.

**Figure 4:**
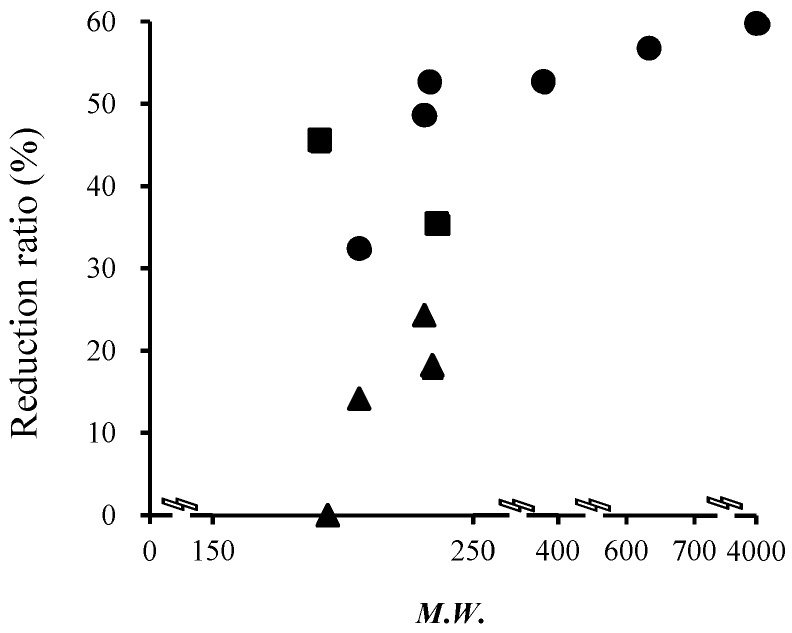
Relationship between the reduction ratio of skin permeation by hair follicle plugging and the molecular weight of the chemicals. ●: ionized form (acidic or basic) chemicals; ▲: non-ionized form (acidic or basic) chemicals; ■: neutral chemicals.

Figure 4 shows the relationship between the molecular weight of topically applied chemicals and the reduction ratio of *p* values by hair follicle plugging. A reduction ratio of *p* values of around 50% was observed for chemicals with a molecular weight greater than 400 Da. The reduction ratio of *p* values seems to increase with an increase in the molecular weight in the range from 200 to 400 Da. However, a dramatic reduction in *p* value was found with an increase in molecular weight from 200 to 400 Da, although the relationship between the reduction ratio of the *p* value of chemicals and their molecular weights was inconsistent, especially from 200 to 350 Da.

**Figure 5:**
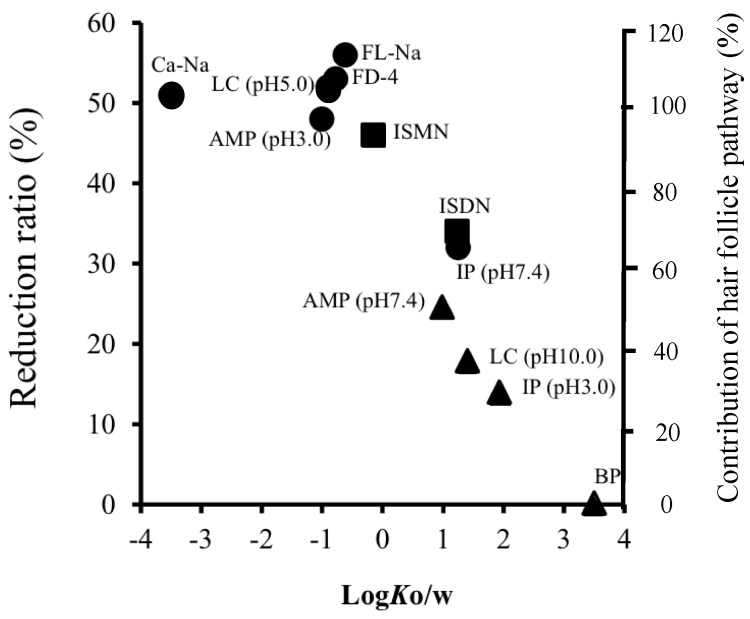
Relationship between the reduction ratio by hair follicle plugging or the contribution of skin permeation pathway and the partition coefficient of the chemicals. ●: ionized form (acidic or basic) chemicals; ▲: non-ionized form (acidic or basic) chemicals; ■: neutral chemicals.

Next, the effect of the lipophilicity of chemicals, log *K*_o/w_, on the reduction ratio of the *p* value was investigated. Figure 5 shows the relationship between the reduction ratio of the *p* value of chemicals and their log *K*_o/w_ values. For hydrophilic chemicals (log *K*_o/w_ < 0), a higher reduction ratio was observed than for lipophilic ones (log *K*_o/w_ ≥ 0). In addition, the reduction ratio decreased with an increase in log *K*_o/w_. Furthermore, about a 10% reduction of chemical permeation was evenly observed for every 1.0 log *K*_o/w_ increase of chemicals (i.e., every 10-fold increase in *K*_o/w_ value) within the range of log *K*_o/w_ values from 0 to 4.

## 4. Discussion

In our previous study, good linear regression was obtained between the decrease in the skin permeation of a high-molecular-weight chemical, FD-4, and the number of hair follicles plugged [21]. About 58 visible hair follicles were confirmed in an effective skin permeation area of 1.77 cm^2^. The point at which the regression line crossed the *x*-axis showed that plugging of 28 and 58 hair follicles led to decreases in the skin permeation of FD-4 of 50 and 100 percent, respectively. Furthermore, no permeation of FD-4 or FL-Na was observed through three-dimensional cultured human skin models in our previous study [8]. This is because the cultured human skin models have no hair follicles. Thus, the present results strongly suggest that the hair follicle pathway is the primary pathway of FD-4 and FL-Na permeation through the skin. In addition, the contribution of hair follicle for FD-4 and FL-Na was not changed after covering stratum corneum with plugging agent for the corresponding hair follicle plugged area (data not shown). Thirty hair follicles in the effective skin permeation area were plugged in this study to calculate the contribution of the hair follicle pathway to the whole skin permeation of topically applied or exposed chemicals.

Among the present results, only *KL* parameters were decreased by hair follicle plugging, while the values of *DL^−2^* were almost constant after the application of AMP solution at different pH levels, suggesting that the plugging method could enable evaluation of the contribution of the hair follicle pathway to the total skin permeation of chemicals. The fraction of the area of orifice of the hair follicle in a diffusion area should be related to the partition parameter of chemicals when hair follicles are its main permeation route [8]. Therefore, the present plugging method and calculation of skin permeation parameters of *KL* and *DL*^−2^ might be useful for calculating the contribution of hair follicles to the permeation of topically applied or exposed chemicals.

Figure 5 shows the relationship between the contribution of the hair follicle pathway and the partition coefficient of chemicals. The contribution of the hair follicle pathway was also compared with other published data to verify our results. Frum et al. [19] reported the influence of log *K*_o/w_ on hair follicle penetration by an in vitro sandwich method. The percentage contribution by follicles decreased with an increase in the lipophilicity of topically applied or exposed compounds, and the levels of contribution (4%, 2%, 46%, 60%, 58%, 46% and 34% for estradiol (log *K*_o/w_: 2.29), corticosterone (log *K*_o/w_: 1.94), hydrocortisone (log *K*_o/w_: 1.60) and aldosterone (log *K*_o/w_: 1.08), respectively) were almost the same as our calculated values. On the other hand, a downward parabolic relationship was observed for the hydrophilic compounds (58%, 48% and 34% for cimetidine (log *K*_o/w_: 0.40), deoxyadenosine (log *K*_o/w_: 0.40) and adenosine (log *K*_o/w_: −1.05), respectively). These data are very different from our calculated values. Frum et al. [19] calculated the hair follicle contribution from a 28 or 48 h skin permeation experiment. Excess hydration of the stratum corneum may change the skin characteristics by causing swelling and the development of pools of water in the intercellular lamellar region [37]. This might be a reason for the low contribution of the hair follicle pathway for hydrophilic drugs compared with our results. Furthermore, the formulation of topically applied drugs would also affect the contribution of the hair follicle pathway due to variation in the partitioning of chemicals in skin. Liu et al. [17] reported that caffeine (log *K*_o/w_: −0.01) was absorbed through hair follicles within 30 min after topical application in a binary solvent of 30% ethanol and 70% propylene glycol, and that 10.5% to 33.8% of the total amount was absorbed through the hair follicles. This binary solvent could increase the skin permeation of chemicals by causing variation in the fluidity of the stratum corneum lipids. However, a much higher contribution of hair follicles (over 90%) was obtained from aqueous solution in the present study. Thus, differences in the formulation should be an important issue to determine the contribution of hair follicles to the permeation of topically applied or exposed chemicals.

In the present study, the contribution of hair follicle route of topically applied chemicals that calculated with the decrease of permeability coefficient after hair follicle plugging treatment almost corresponded with previous reports. However, since excised skin used in an in vitro study might show a histological reduction in the follicular reservoir via contraction of the elastic fibers surrounding hair follicles, Patzelt et al. [38] speculated that an in vitro skin permeation experiment may not be suitable for determining the contribution of the hair follicle pathway to the total skin permeation of chemicals. Silvia et al. [13] reported that the quantity of drug recovered from hair follicle in vitro experiment condition are likely to underestimate compared with in vivo condition. In contrast, Knorr et al. reported [39] that the follicular reservoir of porcine ear skin remains constant after the removal of the ears from cadavers as it is fixed on an underlying cartilage. Raber et al. [40] reported an excellent in vivo/in vitro correlation in terms of the level of nanoparticles recovered from hair follicles after their topical application. In vitro skin permeation experiments have already been broadly used to evaluate and develop transdermal drug delivery systems and to evaluate the risk of skin exposure to chemicals. The contribution of reduced hair follicular-reservoir for the skin permeation of chemicals has not been fully clarified. In addition, skin permeation pathway of chemicals after its distribution into hair follicle route has not been well investigated. Furthermore, there are ethical issues associated with in vivo experiments. Therefore, in the present study, an in vitro experiment was carried out to determine the contribution of the hair follicle pathway. This report reveals that the contribution of the hair follicle pathway to the total skin permeation of topically applied or exposed chemicals could be obtained by hair plugging method with excised skin and be calculated by using lipophilicity of chemicals. Skin permeation parameters *KL* and *DL*^−2^ might be useful to extrapolate skin permeation of chemicals with different hair follicle density sites. Furthermore, experiments should be done to clarify the usefulness of this in vitro approach by comparing in vivo and in vitro data and the observed and the calculated value of skin permeation of chemicals with different hair follicle densities.

## 5. Conclusions

This study shows that the contribution of the hair follicle pathway to the total skin permeation of chemicals can be determined by using their lipophilicity. Thus, consideration of the hair follicle pathway should be considered to investigate the efficacy and safety of chemicals after their topical application or exposure to them because skin permeation and disposition should vary among skins in different body sites due to differences in the density of hair follicles.

## Figures and Tables

**Table 1:** Physicochemical properties of drugs used.

Model Drug	Molecular Weight (*M*_W_)	Log *K*_o/w_ (pH)	p*K*_a_
FD-4	*ca*. 4000	−0.77 (7.4) ^(e)^	6.7 ^(h)^
Ca-Na	644.5	−3.50 (7.4) ^(f)^	5.5 ^(i)^
FL-Na	376.3	−0.61 (7.4) ^(e)^	6.4 ^(e)^
ISDN	236.1	1.23 (7.4) ^(g)^	-
LC	234.3	−0.90 (5.0) ^(b)^ 1.40 (10.0) ^(d)^	7.9 ^(j)^
AMP	231.3	−1.00 (3.0) ^(a)^ 0.98 (7.4) ^(c)^	5.0 ^(k)^
IP	206.3	1.93 (3.0) ^(a)^ 1.25 (7.4) ^(c)^	4.9 ^(l)^
BP	194.2	3.50 (7.4) ^(c)^	8.3 ^(m)^
ISMN	191.1	−0.15 (7.4) ^(g)^	-

^(a)^ *n*-octanol/pH 3.0 citrate buffer log *K*_o/w_ at 32 °C; ^(b)^ *n*-octanol/pH 5.0 citrate buffer log *K*_o/w_ at 32 °C; ^(c)^ *n*-octanol/pH 7.4 phosphate buffer log *K*_o/w_ at 32 °C; ^(d)^ *n*-octanol/pH 10.0 carbonate buffer log *K*_o/w_ at 32 °C; ^(e)^ *n*-octanol/pH 7.4 phosphate buffer log *K*_o/w_ at 32 °C [8]; ^(f)^ *n*-octanol/pH 7.4 phosphate buffer log *K*_o/w_ at 32 °C [25]; ^(g)^ *n*-octanol/pH 7.4 phosphate buffer log *K*_o/w_ at 32 °C [26]; ^(h)^ p*K*_a_ at 25 °C [27]; ^(i)^ p*K*_a_ at 25 °C [28]; ^(j)^ p*K*_a_ at 25 °C [29]; ^(k)^ p*K*_a_ at 25 °C [30]; ^(l)^ p*K*_a_ at 25 °C [31]; ^(m)^ p*K*_a_ at 25 °C [32].

**Table 2:** Skin permeation parameters of ISDN from solutions with different pH.

Skin Permeation Parameter	pH 3.0	pH 7.4	pH 10.0
*Q*_6_ (μmol/cm^2^) ^#^	1.30 × 10^−1^ ± 1.64 × 10^−2^	1.19 × 10^−1^ ± 2.18 × 10^−2^	1.22 × 10^−1^ ± 1.11 × 10^−2^
*P* (cm/s)	2.46 × 10^−6^ ± 4.67 × 10^−7^	2.01 × 10^−6^ ± 2.93 × 10^−7^	1.86 × 10^−6^ ± 2.34 × 10^−7^
*t*_lag_ (h)	1.91 ± 0.23	2.22 ± 0.16	2.02 ± 0.21
*DL^−2^* (cm^−1^)	1.02 × 10^−1^ ± 1.25 × 10^−2^	7.56 × 10^−2^ ± 5.36 × 10^−3^	8.42 × 10^−2^ ± 8.27 × 10^−3^
*KL* (cm)	2.35 × 10^−5^ ± 2.74 × 10^−6^	2.62 × 10^−5^ ± 2.24 × 10^−6^	2.30 × 10^−5^ ± 5.03 × 10^−6^

^#^ *Q*_6_: Cumulative amount of ISDN that permeated through the skin over 6 h. *P*: permeability coefficient; *t*_lag_: lag time; *KL*: partition parameter; *DL^−2^*: diffusion parameter.

**Table 3:** Skin permeation parameters and reduction ratio of AMP with or without hair follicle-plugged skin.

Skin Permeation Parameter at Different pH		Non-PA Plugging	PA Plugging	Ratio ^#^
pH 3.0	*Q*_10_ (μmol/cm^2^)	2.40 × 10^−2^ ± 7.55 × 10^−3^	1.17 × 10^−2^ ± 4.41 × 10^−3^	0.49
	*P* (cm/s)	8.70 × 10^−9^ ± 2.38 × 10^−9^	4.73 × 10^−9^ ± 2.02 × 10^−9^	0.54
	*t*_lag_ (h)	2.53 ± 0.50	2.56 ± 0.40	1.01
	*DL^−2^* (h^−1^)	1.17 × 10^−4^ ± 2.32 × 10^−5^	1.19 × 10^−4^ ± 1.86 × 10^−5^	1.02
	*KL* (cm)	7.58 × 10^−5^ ± 2.00 × 10^−5^	3.99 × 10^−5^ ± 1.70 × 10^−6^	0.52
pH 7.4	*Q*_10_ (μmol/cm^2^)	3.78 × 10^−2^ ± 1.41 × 10^−3^	2.73 × 10^−2^ ± 2.11 × 10^−3^	0.72
	*P* (cm/s)	1.61 × 10^−7^ ± 8.10 × 10^−9^	1.43 × 10^−7^ ± 1.61 × 10^−8^	0.89
	*t_lag_* (h)	3.85 ± 0.14	4.18 ± 0.24	1.09
	*DL^−2^* (h^−1^)	1.78 × 10^−4^ ± 6.34 × 10^−6^	1.94 × 10^−4^ ± 1.11 × 10^−5^	1.09
	*KL* (cm)	9.06 × 10^−4^ ± 5.05 × 10^−5^	7.51 × 10^−4^ ± 1.05 × 10^−4^	0.83

Ratio ^#^: PA plugging/non-PA plugging.

**Table 4:** Reduction in permeability coefficient of chemicals through the HF-plugged skin. Values are the mean ± S.E. (*n* = 3–4).

Chemical	*p* Values through HF-non-Plugged Skin (×10^−8^ cm/s)	*p* Values through HF-Plugged Skin (×10^−8^ cm/s)	Reduction Ratio (%)
FD-4	0.064 ± 0.032	0.026 ± 0.012	59
Ca-Na	2.8 ± 1.2	1.4 ± 1.7	50
FL-Na	1.4 ± 0.57	0.62 ± 0.089	56
ISDN	371 ± 122	242 ± 65	35
Ionized LC	2.3 ± 0.61	1.1 ± 0.31	52
Non-ionized LC	255 ± 109	210 ± 49	18
Ionized AMP	0.88 ± 0.48	0.46 ± 0.42	48
Non-ionized AMP	17 ± 0.74	13 ± 0.50	24
Non-ionized IP	1520 ± 270	1300 ± 81	14
Ionized IP	87 ± 16	59 ± 5.4	32
Non-ionized BP	132 ± 50	132 ± 143	0
ISMN	8.9 ± 1.2	4.9 ± 3.6	45

## References

[1] Naik A., Kalia Y., Guy R. (2000). Transdermal drug delivery: Overcoming the skin’s barrier function. Pharm. Sci. Technol. Today.

[2] Pappas A. (2009). Epidermal surface lipids. Dermato-endocrinology.

[3] Mizutani Y., Mitsutake S., Tsuji K., Kihara A., Igarashi Y. (2009). Ceramide biosynthesis in keratinocyte and its role in skin function. Biochimie.

[4] Crank J. (1979). The Mathematics of Diffusion.

[5] Scheuplein R.J. (1967). Mechanism of percutaneous absorption. II. Transient diffusion and the relative importance of various routes of skin penetration. J. Investig. Dermatol..

[6] Mitragotri S. (2003). Modeling skin permeability to hydrophilic and hydrophobic solutes based on four permeation pathways. J. Control. Release.

[7] Wosicka H., Cal K. (2010). Targeting to the hair follicles: Current status and potential. J. Dermatol. Sci..

[8] Todo H., Kimura E., Yasuno H., Tokudome Y., Hashimoto F., Ikarashi Y., Sugibayashi K. (2010). Permeation pathway of macromolecules and nanospheres through skin. Biol. Pharm. Bull..

[9] Feldman R.J., Maibach H.I. (1967). Regional variation in percutaneous penetration of ^14^C cortisol in man. J. Investig. Dermatol..

[10] Maibach H.I., Feldman R.J., Milby T.H., Serat W.F. (1971). Regional variation in percutaneous penetration in man. Arch. Environ. Health.

[11] Hueber F., Wepierre J., Schaefer H. (1992). Role of transepidermal and transfollicular routes in percutaneous absorption of hydrocortisone and testosterone: In vivo study in the hairless rat. Skin Pharmacol. Physiol..

[12] Grice J.E., Ciotti S., Weiner N., Lockwood P., Cross S.E., Roberts M.S. (2010). Relative uptake of minoxidil into appendages and stratum corneum and permeation through human skin in vitro. J. Pharm. Sci..

[13] Tampucci S., Burgalassi S., Chetoni P., Lenzi C., Pirone A., Mailland F., Caserini M., Monti D. (2014). Topical formulations containing finasteride. Part II: Determination of finasteride penetration into hair follicles using the differential stripping technique. J. Pharm. Sci..

[14] Ogiso T., Shiraki T., Okajima K., Tanino T., Iwaki M., Wada T. (2002). Transfollicular drug delivery: Penetration of drugs through human scalp skin and comparison of penetration between scalp and abdominal skins in vitro. J. Drug Target..

[15] Lieb L.M., Liimatta A.P., Bryan R.N., Brown B.D., Krueger G.G. (1997). Description of the intrafollicular delivery of large molecular weight molecules to follicles of human scalp skin in vitro. J. Pharm. Sci..

[16] Alvarez-Román R., Naik A., Kalia Y.N., Guy R.H., Fessi H. (2004). Skin penetration and distribution of polymeric nanoparticles. J. Control. Release.

[17] Liu X., Grice J.E., Lademann J., Otberg N., Trauer S., Patzelt A., Roberts M.S. (2011). Hair follicles contribute significantly to penetration through human skin only at times soon after application as a solvent deposited solid in man. Br. J. Clin. Pharmacol..

[18] Essa E.A., Bonner M.C., Barry B.W. (2002). Human skin sandwich for assessing shunt route penetration during passive and iontophoretic drug and liposome delivery. J. Pharm. Pharmacol..

[19] Frum Y., Bonner M.C., Eccleston G.M., Meidan V.M. (2007). The influence of drug partition coefficient on follicular penetration: In vitro human skin studies. Eur. J. Pharm. Sci..

[20] Monti D., Tampucci S., Burgalassi S., Chetoni P., Lenzi C., Pirone A., Mailland F. (2014). Topical formulations containing finasteride. Part I: In vitro permeation/penetration study and in vivo pharmacokinetics in hairless rat. J. Pharm. Sci..

[21] Horita D., Yoshimoto M., Todo H., Sugibayashi K. (2014). Analysis of hair follicle penetration of lidocaine and fluoresce in isothiocyanate-dextran 4 kDa using hair follicle-plugging method. Drug Dev. Ind. Pharm..

[22] Otberg N., Patzelt A., Rasulev U., Hagemeister T., Linscheid M., Sinkgraven R., Sterry W., Lademann J. (2008). The role of hair follicles in the percutaneous absorption of caffeine. Br. J. Clin. Pharmacol..

[23] Trauer S., Patzelt A., Otberg N., Knorr F., Rozycki C., Balizs G., Büttemeyer R., Linscheid M., Liebsch M., Lademann J. (2009). Permeation of topically applied caffeine through human skin—A comparison of in vivo and in vitro data. Br. J. Clin. Pharmacol..

[24] Takeuchi H., Takeuchi H., Ishida M., Todo H., Urano H., Sugibayashi K. (2011). Influence of skin thickness on the in vitro permeabilities of drugs through Sprague-Dawley rat or Yucatan micropig skin. Biol. Pharm. Bull..

[25] Yamada K., Yamashita J., Todo H., Miyamoto K., Hashimoto S., Tokudome Y., Hashimoto F., Sugibayashi K. (2011). Preparation and evaluation of liquid-crystal formulations with skin-permeation-enhancing abilities for entrapped drugs. J. Oleo. Sci..

[26] Sugibayashi K., Hayashi T., Matsumoto K., Hasegawa T. (2004). Utility of a three-dimensional cultured human skin model as a tool to evaluate the simultaneous diffusion and metabolism of ethyl nicotinate in skin. Drug Metab. Pharmacokinet..

[27] Cole L., Coleman J., Evans D., Hawes C. (1990). Internalisation of fluorescein isothiocyanate and fluorescein isothiocyanate-dextran by suspension-cultured plant cells. J. Cell Sci..

[28] Heger M., Salles I.I., Van Vuure W., Deckmyn H., Beek J.F. (2009). Fluorescent labeling of platelets with polyanionic fluorescein derivatives. Anal. Quant. Cytol. Histol..

[29] Cázares-Delgadillo J., Naik A., Kalia Y.N., Quintanar-Guerrero D., Ganem-Quintanar A. (2005). Skin permeation enhancement by sucrose esters: a pH-dependent phenomenon. Int. J. Pharm..

[30] Kolthoff I.M., Stenger V.A. (1942). Volumetric Analysis.

[31] Domańska U., Pobudkowska A., Pelczarska A., Gierycz P. (2009). p*K*a and solubility of drugs in water, ethanol, and 1-octanol. J. Phys. Chem. B..

[32] Błędzkaa D., Gryglikb D., Millera S.J. (2009). Photodegradation of butylparaben in aqueous solutions by 254 nm irradiation. J. Photochem. Photobiol. A.

[33] Ahmed S., Imai T., Otagiri M. (1996). Evaluation of stereoselective transdermal transport and concurrent cutaneous hydrolysis of several ester prodrugs of propranolol: Mechanism of stereoselective permeation. Pharm. Res..

[34] Hashida M., Okamoto H., Sezaki H. (1988). Analysis of drug penetration through skin considering donor concentration decrease. J. Pharmacobiodyn..

[35] Okamoto H., Hashida M., Sezaki H. (1988). Structure-activity relationship of 1-alkyl- or 1-alkenylazacycloalkanone derivatives as percutaneous penetration enhancers. J. Pharm. Sci..

[36] Sato K., Oda T., Sugibayashi K., Morimoto Y. (1988). Estimation of blood concentration of drugs after topical application from in vitro skin permeation data. I. Prediction by convolution and confirmation by deconvolution. Chem. Pharm. Bull..

[37] Warner R.R., Stone K.J., Boissy Y.L. (2003). Hydration disrupts human stratum corneum ultrastructure. J. Investig. Dermatol..

[38] Patzelt A., Richter H., Buettemeyer R., Huber H.J., Blume-Peytavi U., Sterry W., Lademann J. (2008). Differential stripping demonstrates a significant reduction of the hair follicle reservoir in vitro compared to in vivo. Eur. J. Pharm. Biopharm..

[39] Knorr F., Patzelt A., Richter H., Schanzer S., Sterry W., Lademann J. (2013). Approach towards developing a novel procedure to selectively quantify topically applied substances in the hair follicles of the model tissue porcine ear skin. Exp. Dermatol..

[40] Raber A.S., Mittal A., Schäfer J., Bakowsky U., Reichrath J., Vogt T., Schaefer U.F., Hansen S., Lehr C.M. (2014). Quantification of nanoparticle uptake into hair follicles in pig ear and human forearm. J. Control. Release.

